# Diffuse leptomeningeal glioneuronal tumour (DLGNT) in children: the emerging role of genomic analysis

**DOI:** 10.1186/s40478-021-01248-w

**Published:** 2021-09-07

**Authors:** Neevika Manoharan, Pamela Ajuyah, Akanksha Senapati, Marie Wong, Anna Mullins, Michael Rodriguez, Helen Doyle, Geoff McCowage, Loretta M. S. Lau, Paul G. Ekert, David S. Ziegler

**Affiliations:** 1 Kids Cancer Centre, Sydney Children’s Hospital, Randwick, NSW 2031 Australia; 2 Children’s Cancer Institute, Lowy Cancer Centre, UNSW Sydney, Kensington, NSW Australia; 3 Cancer Centre for Children, The Children’s Hospital Westmead, Westmead, NSW 2145 Australia; 4 Department of Anatomical Pathology, NSW Health Pathology, Prince of Wales Hospital, Randwick, NSW 2031 Australia; 5 Department of Histopathology, The Children’s Hospital At Westmead, Westmead, NSW 2145 Australia; 6 Murdoch Children’s Research Institute, Royal Children’s Hospital, Parkville, VIC 3052 Australia; 7 School of Women’s and Children’s Health, UNSW Sydney, Kensington, NSW Australia; 8 Cancer Immunology Program, Peter MacCallum Cancer Centre, Melbourne, 3000 Australia; 9 Sir Peter MacCallum Department of Oncology, The University of Melbourne, Parkville, 3052 Australia

**Keywords:** Diffuse leptomeningeal glioneuronal tumour, Paediatrics, Brain tumour, Childhood malignancy

## Abstract

Diffuse leptomeningeal glioneuronal tumours (DLGNT) represent rare enigmatic CNS tumours of childhood. Most patients with this disease share common radiological and histopathological features but the clinical course of this disease is variable. A radiological hallmark of this disease is widespread leptomeningeal enhancement that may involve the entire neuroaxis with predilection for the posterior fossa and spine. The classic pathologic features include low- to moderate-density cellular lesions with OLIG2 expression and evidence of ‘oligodendroglioma-like’ appearance. The MAPK/ERK signaling pathway has recently been reported as a potential driver of tumourigenesis in up to 80% of DLGNT with *KIAA1549:BRAF* fusions being the most common event seen. Until now, limited analysis of the biological drivers of tumourigenesis has been undertaken via targeted profiling, chromosomal analysis and immunohistochemistry. Our study represents the first examples of comprehensive genomic sequencing in DLGNT and shows that it is not only feasible but crucial to our understanding of this rare disease. Moreover, we demonstrate that DLGNT may be more genomically complex than single-event MAPK/ERK signaling pathway tumours.

**Supplementary Information:**

The online version contains supplementary material available at 10.1186/s40478-021-01248-w.

## Introduction

Diffuse leptomeningeal glioneuronal tumours (DLGNT) are rare central nervous system (CNS) tumours defined in the 2016 World Health Organisation (WHO) classification of CNS neoplasms [39]. We report on two molecularly-distinct cases of DLGNT that represent the only patients in the literature to our knowledge whom have been sequenced using a comprehensive molecular profiling platform including whole genome sequencing (WGS) of germline and tumour DNA, transcriptome analysis (RNAseq) and DNA methylation profiling (based on the Epic 850 K array) [8]. These cases emphasise the importance of identifying the genomic and epigenomic drivers of tumourigenesis in DLGNT and other rare CNS tumours of childhood [14, 16, 49].

## Case presentation

### Case 1

**Fig. 1:**
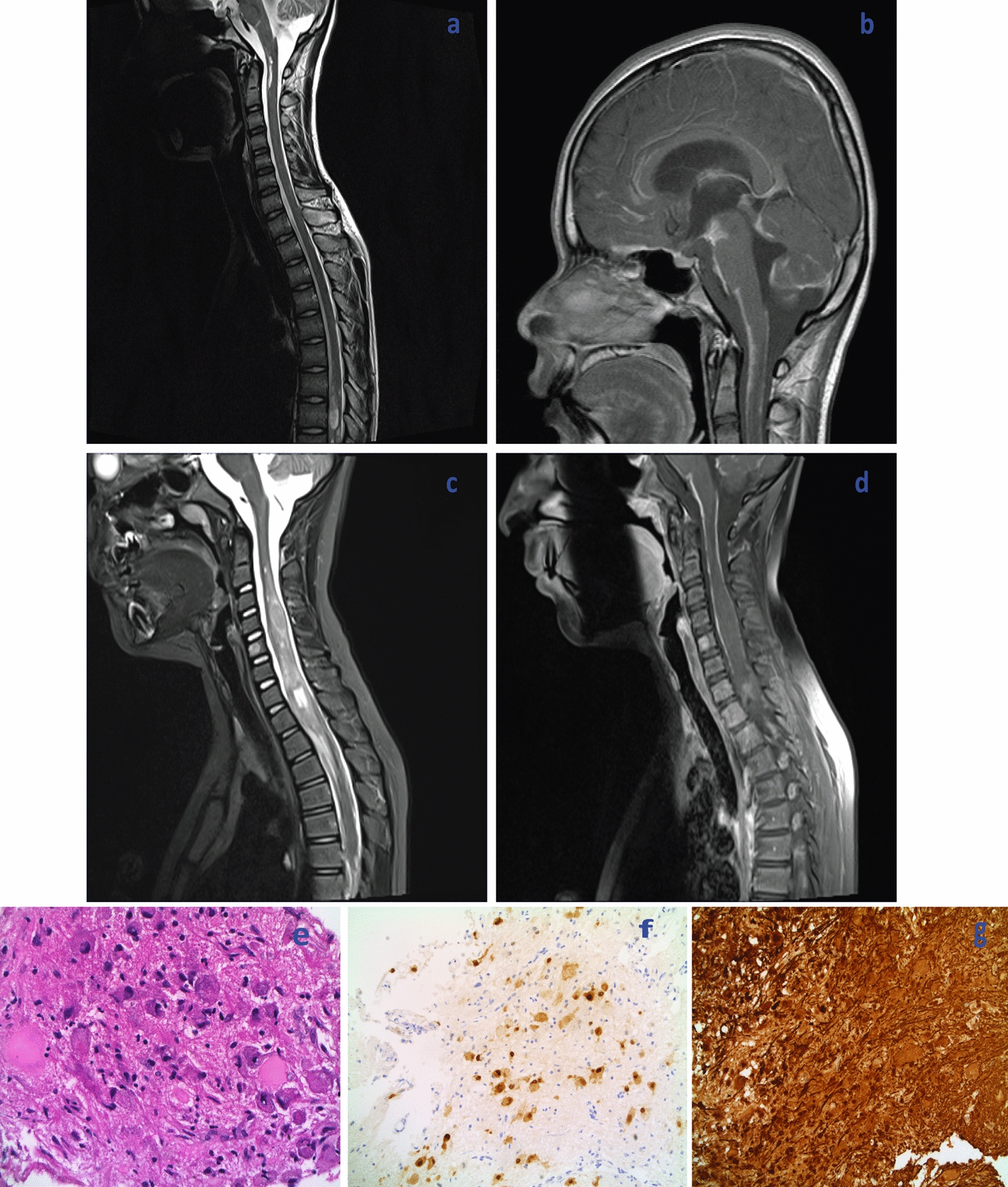
Radiological features of DLGNT on sagittal, T2-weighted images (**a**, **c**), T1 post-gadolinium imaged (**b**, **d**) and histopathological features of DLGNT (**e**, **f**, **g**): Radiology demonstrating **a** intramedullary C1-2 and T7-9 lesions. **b** ‘sugar coating’ leptomeningeal spread with nodular disease at cavernous sinus. **c** Intramedullary C2-T1 lesion with solid and cystic components. **d** ‘sugar coating’ leptomeningeal spread. Pathology of intramedullary tumour of case 2 demonstrating e. high power of H&E sections (400×) of dysplastic ganglionic type neurons with admixed neoplastic glial cells, **f** Neu-N positive dysplastic ganglionic type neurons, **g** diffuse positive staining of GFAP

A 13-year-old boy presented with a one-week history of lethargy, headaches and nausea. At presentation he was noted to be drowsy and had a left sided facial droop, left sided weakness and dysarthria. An MRI showed multiple foci of abnormal T2 hyperintensity in the anterior spinal cord at C2 and large areas of abnormal T2 hyperintensity within the cord at T7-9, all of which were contrast-enhancing (Fig. 1a, b). Although there were no risk factors for tuberculosis and CSF, serum and urine were negative for acid fast bacilli and mycobacteria, anti-TB, anti-bacteria and antiviral treatments were commenced based on the MRI findings. CSF cytology was negative for tumour cells and had negative tumour markers.

Biopsy of a cavernous sinus lesion was performed and the post-operative course was complicated by recurrence of hydrocephalus requiring an additional external ventricular drain and ultimately the insertion of a ventriculoperitoneal shunt. The biopsy was small and crushed but showed small ovoid OLIG-2 positive cells with hyperchromatic nuclei in prominent myxoid stroma. There was insufficient material for flow cytometry. FISH was negative for 1p and 19q loss. Ultimately the histopathology was inconclusive and a definitive diagnosis could not be determined.

A sample was submitted for molecular profiling to the Zero Childhood Cancer (ZERO) national personalised medicine program to assist with the diagnostic process and clinical management [58]. Samples enrolled into the ZERO national trial (PRecISion Medicine for Children with Cancer study—PRISM) require both tumour and matching germline samples from patients with high-risk paediatric cancer. Due to diagnostic uncertainty, and persisting MRI changes 1 month after initial presentation, consideration was given to further biopsy. However, the genomic analysis from the PRISM study resulted in the diagnosis of DLGNT and prevented the need for further invasive surgical intervention.

**Fig. 2:**
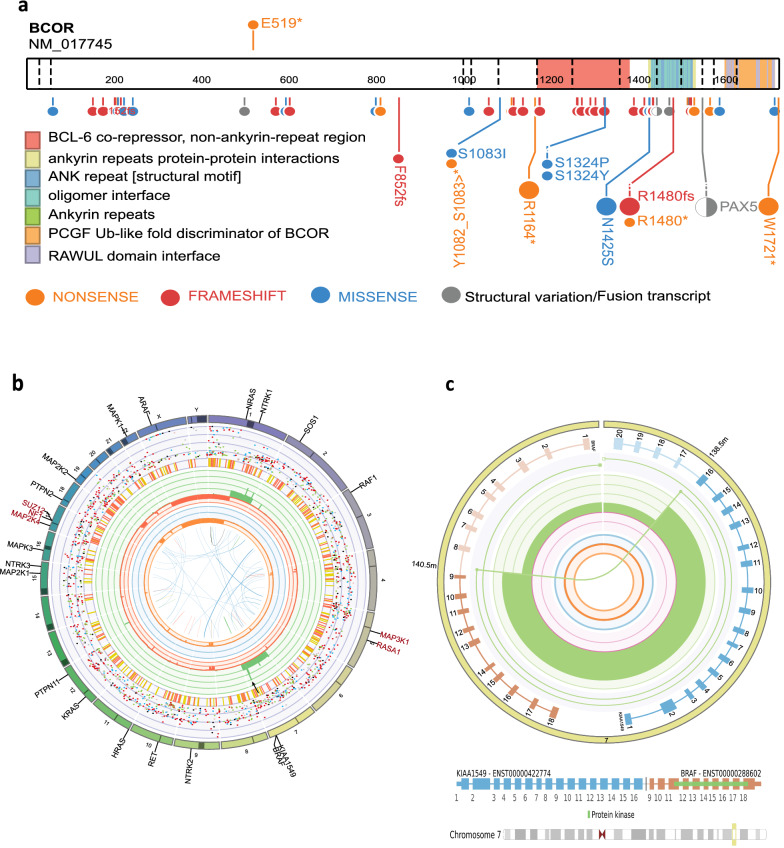
Molecular alterations within DLGNT case 1. **a** Schematic of the BCOR gene demonstrating protein domains and the location of the E519* variant. Synonymous mutations have been excluded and data from PeCan incorporated. **b** CIRCOS plot illustrating the somatic alterations in the tumour and labelling of common tumour suppressor genes (brown) and oncogenes (black) in the MAPK pathway. The CIRCOS plot can be interpreted as follows: The outer first circle demonstrates the 22 autosomal chromosomes and the sex chromosomes. The dark band within the chromosome represents the centrosomes with the p arm to the left of the band and the q arm to the right. The dark bands can also be representative of heterochromatin or missing p arms. The following circle (light purple) illustrates the somatic single nucleotide variants with each dot representing a missense change (C > A blue, C > G black, C > T red, T > A grey, T > A grey, T > C green, T > G pink) and the location of the dot within the circle is associated with its corrected allele frequency (0% at the bottom edge of the ring to 100% at the top edge of the ring). The next ring consists of short insertions and deletions (yellow and red, respectively). The third circle consisting of red and green shading shows the copy number alterations in the tumour with red indicating loss and green indicating gains/amplifications (with the scale ranging from a complete loss of 0 up to gains and above of 6). The fourth circle (orange and blue shading) demonstrates the ‘minor allele copy numbers’ ranging from 0 to 3. Loss of heterozygosity is indicated in orange and is for values below 1, whereas amplification of both alleles is shown in blue and it will be any value above 1. The innermost circle represents different types of structural variants in the tumour (translocations (blue), deletions (red), insertions (yellow), tandem duplications (green) and inversions (black). **c** LINX plot demonstrating the KIAA1549-BRAF dup in this case. The outer edge shows the chromosomes affected by structural variants (SVs) in the tumour and the position of their breakends. The KIAA1549 and BRAF genes are shown in blue and brown, respectively with their exons numbered. The internal circle shows the derivative chromosome segments with breakends shown as shaded circles and the same event united with the same coloured line. The inner green and pink circles show copy number gain or loss, respectively and the inner blue and orange circles show the minor allele ploidy. The innermost circle demonstrates the break junctions of the SVs

Molecular profiling of the cavernous sinus biopsy demonstrated multiple somatic genetic findings and no reportable germline findings (summarized in Table 1). A somatic pathogenic nonsense BCOR variant (p.Glu519Ter) was identified in the tumour. BCOR is a transcriptional repressor that plays a role in chromatin remodeling and acts as a tumour suppressor gene [5]. The p.Glu519Ter variant is a truncating mutation in exon 4/15 creating a premature stop codon predicted to result in an absence of protein due to nonsense mediated decay or complete loss of function due to an aberrant protein product. BCOR is situated on the X chromosome and being that this is a male patient, the hemizygous mutation would result in no functional copies. The p.Glu519Ter variant is not present in population databases and in silico tools predict the mutation to be damaging. Furthermore, most pathogenic/likely pathogenic mutations in the BCOR gene within the ClinVar and PeCan database are loss of function mutations, providing additional support to the pathogenicity of this variant [18, 60] (Fig. 2a). Interestingly, the study by Deng et al. examining histopathological and molecular alterations in a DLGNT cohort also identified a loss of function BCOR variant [14]. Molecular findings such as these emphasise the importance of exploring the genomic landscape of these rare tumours with NGS.

**Table 1:** Molecular profiling of DLGNT cases from the PRISM clinical trial

	Case 1	Case 2
Tumour purity %	71	79
Ploidy	Diploid	Diploid
Tumour mutational burden (mutations/Mb)	0.68 (low)	0.98 (low)
Mutational signatures	NIL	NIL
Somatic mutations	BCOR:c.1555G > T (p.Glu519Ter)	RET:c.2755G > C (p.Ala919Pro)
Fusions	KIAA1549-BRAF	KIAA1549-BRAF
Reportable copy number alterations	1p/19q loss, 1q gain	1p loss, 1q gain, chr8 gain
Germline mutations	NIL	NIL
RNA Expression	Degraded sample	Insufficient sample
Methylation	Match: methylation class diffuse leptomeningeal glioneuronal tumor (0.97)	No match: methylation class family Glioblastoma, IDH wildtype (0.78)

Molecular alterations from WGS, RNAseq and methylome data as curated and discussed at a Multidisciplinary Tumour Board

Analysis of copy number variants revealed a 1p/19q co-deletion and 1q gain which is in keeping with a subclass of DLGNTs (Fig. 2b). Additional copy number changes of unclear significance were gain of chromosome 7 and focal losses in the 9p arm (Additional file 1: Tables 1 and 2). A KIAA1549-BRAF fusion was also identified (Fig. 2c). A methylation array is part of the ZERO profiling of CNS tumours, and is primarily used as an orthogonal diagnostic technique. Raw IDAT files were processed through the DKFZ Molecular Neuropathology (MNP) 2.0 classifier and the sample was a match with diffuse leptomeningeal glioneuronal tumour (probability = 0.97). The combination of the copy number changes, the KIAA1549-BRAF fusion and methylation classifier result aided in resolving this clinical diagnostic dilemma.

Treatment was commenced with a combination regimen of vincristine and carboplatin. After 6 months of treatment there was reduction in size of the intramedullary lesions and stabilisation of the leptomeningeal disease. However, the patient experienced an anaphylactic reaction to carboplatin and was subsequently treated with 6 months of TPCV (thioguanine, procarbazine, lomustine and vincristine) and achieved disease stability throughout treatment. The patient has now completed treatment and has no clinical or radiological evidence of disease progression 16 months from diagnosis.

### Case 2

An 8-year-old girl presented with acute disorientation with associated vomiting and headache. There was a background history of intermittent, self-resolving headaches over the prior 3–4 months. She was noted to have moderate to severe papilloedema with associated haemorrhages, engorged vessels and decreased visual acuity. MRI brain and spine demonstrated a mass in the spinal cord extending from C3 to T2, filling most of the spinal canal. There was some associated T2 hyperintensity with several areas of cyst formation or necrosis and an enhancing nodule at the cervicothoracic junction (Fig. 1c, d).

The initial biopsy of a meningeal deposit was inconclusive. A second biopsy of the intramedullary tumour was performed. Histopathology showed features of a low grade tumour with both glial and neuronal differentiation (Fig. 1e–g). There were neoplastic glial cells and dysplastic neuronal cells with a ganglionic appearance. Immunohistochemistry staining for BRAF V600E mutation was negative. Chromosome microarray detected a 1p deletion, 1q gain, chromosome 17 gain and indicated a KIAA1549-BRAF fusion. A histopathological diagnosis of DLGNT was made based on the above findings.

**Fig. 3:**
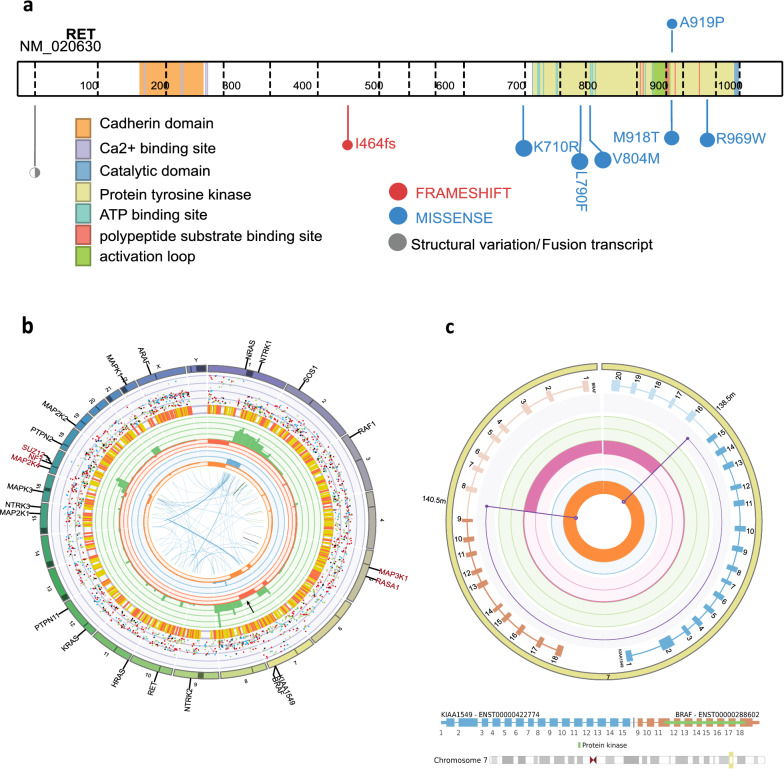
Molecular alterations within DLGNT case 2. **a** Schematic of the RET gene demonstrating protein domains and the location of the A919P variant in this tumour along with the most commonly seen missense variant M918T (COSMIC) and mutations from PeCan. **b** CIRCOS plot indicating various copy number alterations from the tumour in case 2. **c** LINX plot demonstrating the complex KIAA1549-BRAF dup in case 2

Molecular profiling of a lesional biopsy sample through PRISM identified a somatic RET variant of unknown significance (p.Ala919Pro). RET is a receptor tyrosine kinase that is an upstream member of the MAPK pathway [33]. This missense variant is situated in an ATP binding site and the protein tyrosine kinase domain 2 and in silico models have predicted this variant to be damaging. It is adjacent to a well-studied pathogenic gain of function mutation Met918Thr [27, 53] (Fig. 3a). Functional studies on the RET A919P variant suggest that this variant has an additive effect but may require a second hit in the RET gene to be a tumourigenic driver [27]. The RET A919P variant along with the inclusion of a downstream pathway member such as the KIAA1549-BRAF fusion (identified in this tumour) may have an amplificative effect on the MAPK pathway. However, without biological confirmation, the RET mutation remains a variant of unknown significance in this tumour.

Copy number analysis revealed an aneuploid tumour with multiple alterations of unknown significance (Fig. 3b, Additional file 1: Tables 3 and 4). However, a loss of chromosome 1p and a gain of chromosome 1q and chromosome 8 were also detected, which are in keeping with a subclass of DLGNT [14]. This tumour also contained a KIAA1549-BRAF fusion with an unknown highly repetitive DNA fragment inserted within the duplication (Fig. 3c). BLAT analysis of the repetitive sequence could not identify with high certainty the origin of this fragment. This can be visualised with the LINX plot in Fig. 3c with single end breakends (the purple lines with open unshaded circles) bookending the KIAA1549-BRAF duplication as the other breakend match within the genome of the tumour could not be established. This adds to the complexity of the formation of the KIAA1549-BRAF fusion within this tumour which also has 1 copy loss of the 7q arm where the KIAA1549 and BRAF genes reside. This emphasizes that very complex genomic rearrangements may underlie the expression of even common fusion drivers. The combination of whole genome and RNA sequencing often helps resolve such complexity [58]. Methylation studies processed through the DKFZ CNS classifier found that the patient’s sample was not a strong match to any particular entity. The closest match was glioblastoma, subclass midline, IDH wildtype (0.78).

Treatment with vincristine and carboplatin was commenced but ceased after 3 cycles due to moderate to severe drug reactions to carboplatin (fever, tachycardia and hypotension). The patient was subsequently treated with trametinib, based on the presence of the KIAA1549-BRAF duplication, with early evidence of minor response on surveillance MRI. Subsequently, the patient had disease progression after 6 months on trametinib. Further treatment options are currently being explored.

## Discussion

Diffuse leptomeningeal glioneuronal tumours are a rare group of central nervous system (CNS) tumours, with less than 100 cases reported in the literature [1, 4, 7, 9, 11, 13, 14, 16, 17, 23–25, 34, 42–47, 49–51]. The majority of cases are reported in children but adult cases have also been described [11, 13, 45]. Although defined in 2016, DLGNT is likely synonymous with other historical entities variably described in the literature prior to the 2016 classification as ‘Primary diffuse leptomeningeal oligodendroglioma’ [24], ‘Diffuse leptomeningeal oligodendrogliomatosis’ [4, 7, 9], ‘Primary disseminated leptomeningeal oligodendroglioma’ [7], ‘Disseminated oligodendroglial-like leptomeningeal tumour of childhood’ [45] and ‘Superficially disseminated glioma of children’ [1]. These tumours are characterized radiologically by widespread leptomeningeal enhancement on MRI and histologically by low to moderate-density with cellular features that are akin to oligodendrogliomas [1, 4, 22, 25, 45]. Attempts to classify this group of tumours as a single pathologic entity were buoyed by Rodriguez et al.’s publication of the largest case series of 36 cases and description of common radiological, histopathological and chromosomal abnormalities demonstrated on FISH and SNP array [45]. Here we demonstrate the findings of the first comprehensive genomic profiling of two cases of DLGNT that further our understanding of this rare and enigmatic disease.

The clinical presentation of patients with DLGNT are varied, dependent on area(s) of disease involvement and range from paraesthesia and seizures to symptoms of hydrocephalus, such as headache and vomiting [1, 11, 16, 45, 49]. The diagnosis of DLGNT can be complicated by the wide range of differential diagnoses for leptomeningeal enhancement on MRI scans including infection and subarachnoid haemorrhage and difficulties in obtaining a histopathological diagnosis due to small biopsy samples [54, 56].

Radiologically, the classic MRI findings in DLGNT include leptomeningeal enhancement involving the brain and spine associated with cystic T2 hyperintense lesions that may not enhance. The intracranial disease is typically most evident in the basal cisterns, Sylvian fissures, brainstem and cerebellar folia. Discrete parenchymal abnormalities are described in the spine and can be associated with thickening of nerve roots [16, 45, 48, 49, 55, 56]. Although multiple cases of DLGNT display this classical appearance on MRI, others are atypical in radiological appearance but consistent with the histopathological and/or molecular diagnosis [47, 50].

The histopathologic diagnosis of DLGNT is often elusive due to limited tumour material from biopsies of sparse leptomeningeal disease and as a result, multiple patients have non-diagnostic initial biopsies [2, 49]. As in Case 1, patients may be empirically commenced on treatment for another condition such as tuberculous meningitis or subarachnoid haemorrhage based on radiological and clinical features [50, 51, 54]. As a result, diagnostic workup can be prolonged and treatment for DLGNT delayed. Patients with cerebrospinal fluid (CSF) sent for cytology typically show no evidence of tumour cells despite extensive leptomeningeal disease radiologically [1, 45, 49]. The classic histopathologic features of DLGNT are that of a low- to moderate-cellularity tumour with monomorphic oligodendroglioma-like cells surrounded by dense collagen. The monomorphic intermediate-sized cells typically have low mitotic activity (median 0–4 mitoses per high power field) and low Ki-67 labelling-index (< 5%) and the majority of patients displayed desmoplastic stroma [45]. OLIG2 expression on IHC is classic with more variable expression of GFAP or synaptophysin [11, 45, 49]. Although the majority of patients have histopathologic features consistent with a low-grade glioneuronal tumour, a small subset have concerning high-grade pathologic features including anaplastic changes, focally elevated proliferative index (up to 53% in some cases) and glomeruloid microvascular changes [34, 41, 45, 50].

The clinical course for the majority of patients with DLGNT is indolent with some patients displaying slow asymptomatic progression without therapy for over 18 months [16, 45, 50]. Unfortunately, for a subset of patients, the clinical course is more aggressive and relentless and results in patients dying of their disease [16, 22, 45]. Due to the rarity of this disease and lack of established treatment protocols, comparison of clinical outcomes for patients on different therapies is not possible [2, 16]. Treatment strategies employed for patients with this disease range from observation only, to chemotherapy, to craniospinal radiation. Multiple paediatric low grade glioma (pLGG) chemotherapy protocols have been administered in this patient population including combinations of vincristine and carboplatin (VCR/carboplatin); carboplatin monotherapy; vinblastine monotherapy; bevacizumab; temozolomide, cisplatin/etoposide and combination therapy with thioguanine, procarbazine, lomustine and vincristine (TPCV) [6, 12, 15, 26, 38, 40]. These treatment protocols have resulted in some patients achieving a partial response (PR) and others achieving long-term stable disease (SD), suggesting a role for pLGG chemotherapy in DLGNT [2, 16, 45, 57]. Other reports have described clear clinical improvement and radiological stability after craniospinal irradiation in a subset of patients with DLGNT [16, 22, 45]. The rarity of DLGNT has precluded the development of randomized clinical trials to determine the optimal treatment of this disease.

Recent advances in genomic analyses of childhood CNS tumours have resulted in an improved understanding of key genetic alterations in multiple types of brain tumours. In DLGNT, these analyses have highlighted the sentinel role of activation of the mitogen-activated protein kinase/extracellular signal regulated kinase (MAPK/ERK) pathway in tumourigenesis [10, 11, 14, 16, 46]. The pivotal role of aberrant MAPK/ERK pathway activation has been well described as a crucial driver in pLGG and targeting this pathway is now the focus of multiple paediatric clinical trials [20, 28, 29, 35–37]. The most frequent alteration described in pLGG is *BRAF* duplication with *KIAA1549* being the most common fusion partner [59]. Recent publications have highlighted the role of the MAPK/ERK pathway alterations in DLGNT with *KIAA1549:BRAF* fusions being the most common genomic event seen with deletion of chromosomal arm 1p or 1p/19q co-deletion [11, 14, 16, 46]. Despite the oligodendroglioma-like appearance of DLGNT on histopathology, IDH mutations are not apparent in this disease [14, 16]. Other alterations described in DLGNT include *BRAF* V600E mutation and fusions of *NTRK1/2/3* and *TRIM33:RAF1*, which are known to result in activation of the MAPK/ERK pathway [3, 14, 16, 30, 31, 59].

Our work and others describe MAPK/ERK pathway alterations as the dominant pathway alteration, identified in up to 66–80% of DLGNT [11, 14, 16, 46]. Despite multiple recent publications highlighting the potential role of MAPK/ERK pathway alterations in DLGNT, many have limited their analysis to targeted profiling of *BRAF* alterations, and FISH and SNP arrays as methods of detecting MAPK/ERK pathway alterations [11, 16, 46]. Through PRISM, we were able to undertake comprehensive profiling of the cancer genome of DLGNT to interrogate the genomics of this rare disease to a depth that has not previously been described in the literature [58]. The PRISM program resulted in a patient receiving a diagnosis when histopathological review alone was unable to diagnose DLGNT and in this patient further pursuit of high-risk surgical biopsy was avoided by comprehensive genomic analysis (Case 1).

Although the comprehensive profiling performed in PRISM is not universally accessible, it has the potential to identify novel drivers of tumourigenesis, which will be crucial in our understanding of this rare disease. In particular, this will be crucial for the 20–30% of patients with DLGNT for whom MAPK/ERK pathway alterations are not found and the oncogenic driver(s) remain elusive. With limited tumour material, we were able to undertake comprehensive profiling including WGS, methylation and germline testing. This allowed for detection of the *KIAA1549:BRAF* fusion in both patients as well as a pathogenic somatic BCOR variant in case 1 and a somatic RET variant in case 2, along with numerous copy number variations that we have not reported on due to uncertain diagnostic and prognostic significance. Intriguingly, the recent publication by Deng et al. study also identified somatic variants in the BCOR and ATRX genes, which are known in the literature to be epigenetic regulators [5, 14].

Targeting the MAPK/ERK pathway in paediatric CNS tumours has been an area of significant progress in the last decade [32, 35]. With the advent of inhibitors of *MEK* and *BRAF*, targeted therapeutic options have been shown in clinical trials to have a role in relapsed/refractory pLGG and paediatric high grade glioma (pHGG) [19, 36]. Although the use of targeted inhibitors of *MEK* and *BRAF* have not yet been well described in DLGNT, demonstrating the sentinel role of the MAPK/ERK pathway will potentially allow these therapeutic options to be used in paediatric patients with DLGNT (eg. case 2) in the future [35–37].

Deng et al. described two separate methylation classes of DLGNT (DLGNT-MC-1 and DLGNT-MC-2) with suggestion that DLGNT-MC-2 encompasses the patients with a poorer prognosis and clinical outcome [8, 14]. The detailed copy number analysis from our study allowed us to segregate the two DLGNT tumours based on the suggested molecular profiles from Deng et al. The genomic profile from case 1 matches well with the DLGNT-MC-1 subclass due to the 1p loss and 7q34 gain associated with both classes, but more specifically the 1p/19q co-deletion observed in 47% of DLGNT-MC-1 vs 15% in DLNT-MC-2. In contrast, case 2 closely resembles DLGNT-MC-2, specifically with the 1q gain (100% of DLGNT-MC-2 vs. 35% DLGNT-MC-1), chromosome 8 gain (54% of DLGNT-MC-2 vs 6% in DLGNT-MC-1) and absence of 1p/19q co-deletion. Furthermore, case 2 contains a focal 7q34 gain (where the KIAA1549-BRAF fusion resides), but equates to a balanced ploidy in a region of copy number loss as the entirety of 7q is haploid. We postulate that potentially the loss of identifiable methylation probes in this region along with the unusual fragment within the KIAA1549-BRAF fusion of probable retrotransposon origin has led to the poor methylation match within the DKFZ CNS classifier for this case.

Our work will contribute to the genomic analysis of DLGNT and the ability of future profiling studies to identify alterations to the epigenome as a feature of DLGNT tumours along with the MAPK pathway. Furthermore, we were able to exclude underlying germline mutations which have not been well studied in this disease and only identified in two previous patients, one with a germline *TP53* variant and another with germline *RAF1* mutation and cardio-facio-cutaneous syndrome [16, 52].

The divergent clinical outcomes of patients with DLGNT remain perplexing, with some patients experiencing indolent chronic disease and others having an aggressive relentless clinical trajectory [16, 21, 45, 49]. Establishing a link between the clinical paradigm and the genomics in these patients, remains an area of significant interest. We reported on two cases with genetic profiles most resembling DLGNT-MC-1 (case 1) and DLGNT-MC-2 (case 2), despite both cases displaying the *KIAA1549-:BRAF* fusion. In addition, our identification of a pathogenic BCOR variant (case 1) and somatic RET variant (case 2) suggests a wider genetic profile for this disease and evidence of intra-tumoral molecular heterogeneity that may contribute to variations in clinical outcomes.

## Conclusions

Diffuse leptomeningeal glioneuronal tumours (DLGNT) are an enigmatic and heterogeneous group of rare CNS tumours that are an indolent disease for some patients and an aggressive fatal tumour for others. With emerging knowledge about the role of MAPK/ERK pathway aberrations in the majority of children with this disease, we highlight the value of comprehensive profiling of cancer genomes in these patients. This profiling assists in diagnosis, allows for detection of other novel genomic alterations that may be oncogenic drivers, and may contribute towards our understanding of the divergent clinical outcomes in this disease.

## Supplementary Information

**Additional file 1.** Supplementary Tables 1 & 2: Single Nucleotide Variants (SNVs) and Copy Number Variants (CNVs) identified in case 1 from WGS, Supplementary Tables 3 & 4: SNVs and CNVs identified in case 2 from WGS

**Additional file 2**: Supplementary Figure 1: Histopathology images from case 1 demonstrating: a. high power (×400) H&E staining demonstrating tumour cells embedded in fibromyxoid stroma, b. OLIG2 stain (×200), c. synaptophysin stain (×400), CD56 stain (×400)

## Data Availability

WGS, RNAseq and methylation data generated by this study are available from the European Genome-phenome Archive under accession number EGAS00001004572. Databases used to help filter, prioritize and interpret variants are available online, including COSMIC (https://cancer.sanger.ac.uk/cosmic), Cancer Gene Census (https://cancer.sanger.ac.uk/census), Pecan (https://pecan.stjude.cloud/), dbscSNV (http://www.liulab.science/dbscsnv.html), dbNSFP (https://sites.google.com/site/jpopgen/dbNSFP), ExAC (http://exac.broadinstitute.org/), gnomAD (https://gnomad.broadinstitute.org/), MGRB (https://sgc.garvan.org.au/), GIAB (https://jimb.stanford.edu/giab-resources), Platinum Genomes (https://github.com/Illumina/PlatinumGenomes), ClinVar (https://www.ncbi.nlm.nih.gov/clinvar/), ESP (https://evs.gs.washington.edu/EVS/) and 1000 Genomes (https://www.internationalgenome.org/data).

## Abbreviations

BCOR: BCL6 corepressor
CSF: Cerebrospinal fluid
CNS: Central nervous system
DLGNT: Diffuse leptomeningeal glioneuronal tumor
FISH: Fluorescence in situ hybridization
HTS: High-throughput drug screen
IDH: Isocitrate dehydrogenase
IHC: Immunohistochemistry
MAPK/ERK: Mitogen-activated protein kinase/ extracellular signal regulated kinase
MRI: Magnetic resonance imaging
OLIG2: Oligodendrocyte transcription factor 2
PDX: Patient-derived xenograft
pHGG: Pediatric high-grade glioma
pLGG: Pediatric low-grade glioma
PR: Partial response
RET: Rearranged during transfection protooncogene
SD: Stable disease
WGS: Whole genome sequencing
WHO: World Health Organisation
