# Supplementary figures and images for: Diffuse leptomeningeal glioneuronal tumour (DLGNT) in children: the emerging role of genomic analysis
Source: Acta Neuropathol Commun. 2021 Sep 7;9:147. doi: 10.1186/s40478-021-01248-w (PMC8422739; doi:10.1186/s40478-021-01248-w)

**a**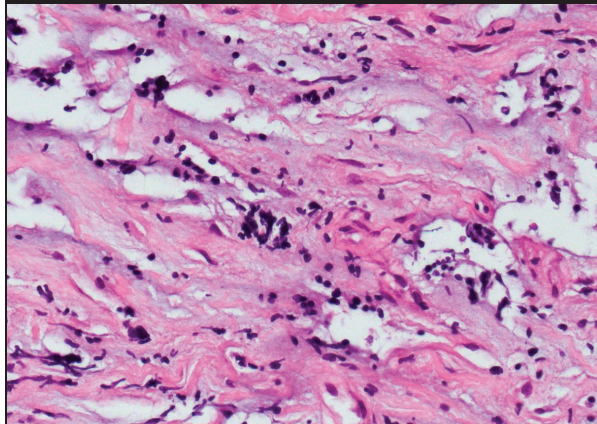**b**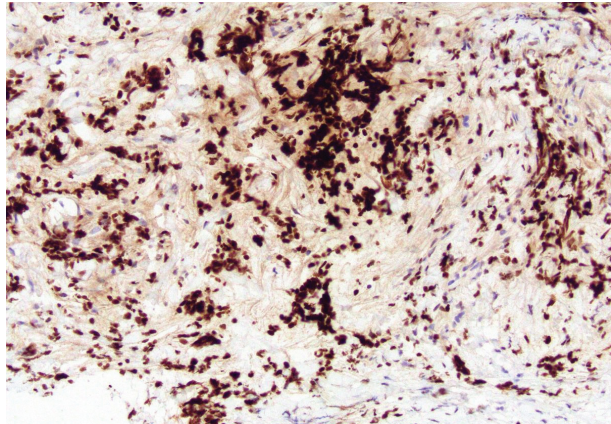**c**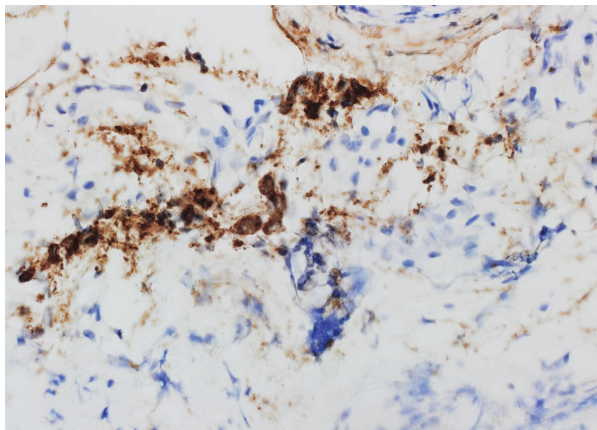**d**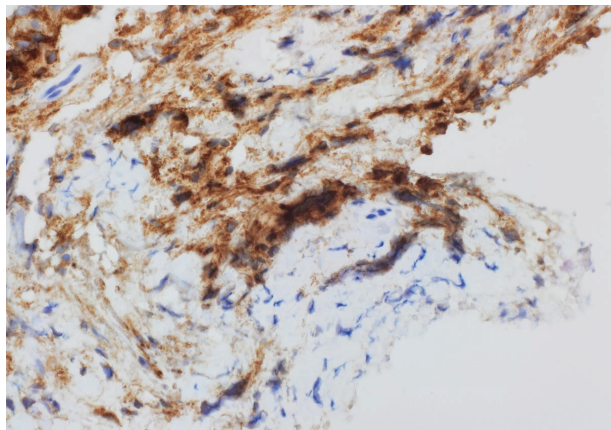

Supplement: Supplementary file 2 — Additional file 2: Supplementary Figure 1: Histopathology images from case 1 demonstrating: a. high power (×400) H&E staining demonstrating tumour cells embedded in fibromyxoid stroma, b. OLIG2 stain (×200), c. synaptophysin stain (×400), CD56 stain (×400) [file 40478_2021_1248_MOESM2_ESM.pdf]
